# Validation of EPICOG-SCH Screening Battery in First Episode Psychosis: Cohort Study to Follow up Cognition and Functionality

**DOI:** 10.1192/j.eurpsy.2025.2283

**Published:** 2025-08-26

**Authors:** R. Ayesa, S. Zaragoza Domingo, J. Bobes, M.-P. García-Portilla, R. Magdalena-Herrero, A. Yorca-Ruiz, M. de Gracia Blanco, G. Escartin, V. Ortíz-García de la Foz, J. Ruiz

**Affiliations:** 1 Vadecilla Research Biomedical Institute, IDIVAL, School of Medicine, University of Cantabria, CIBERSAM, Santander; 2 R&D, Neuropsychological Research Organization s.l (Neuropsynchro), Barcelona; 3 Psychiatry Departmen, 3) Psychiatry Department, University of Oviedo, CIBERSAM; 4 Psychiatry Department, University of Oviedo, Oviedo; 5 Psychology School, University of Girona, Girona; 6 Psychiatry Departmen, Parc Sanitari Sant Joan De Déu, Barcelona; 7 Faculty of Psychology, University of Barcelona, Barcelona, Spain

## Abstract

**Introduction:**

The Epidemiological Study of Cognitive Impairment in Schizophrenia (EPICOG-SCH) is a brief battery to screen for cognitive impact of schizophrenia in outpatient settings. The EPICOG-SCH includes well-known subtests available worldwide that cover key cognitive domains demonstrated to be related to a variety of functional outcomes. Novel composite scores are modelled to predict patient’s functionality in daily life at short, mid and long term.

**Objectives:**

We want to progress in the elaboration of specific algorithms first episodes of schizophrenia at different follow up periods by modelling cognitive performance to predict short, mid and long term functionality.

**Methods:**

Data for the present investigation were obtained from an the epidemiological and three-year longitudinal intervention program of first-episode psychosis (PAFIP) conducted at the outpatient clinic and the inpatient unit at the University Hospital Marques de Valdecilla, Spain. The cohort is composed by 167 patients with a diagnose of Schizophrenia and 160 healthy controls recruited between February 2001 to February 2014. For all analyses, a subset of measures was selected corresponding to the EPICOG-SCH domains with the Rey Auditory Verbal Learning Test (RAVLT) task ifor Logical Memory. Functional outcomes were measured by the Disability Assessment Scale (DAS) and the General Assessment for Function (GAF) scales. Functional measurements were conducted regarding premorbid, baseline set at clinical stabilization and 1, 3 year follow-up, and cognitive assessments where conducted at baseline and 1, 3 years of follow up.

**Results:**

A cohort of 122 patients and 114 Controls completed the study with a 3-year follow up were included in the analysis. Changes across evaluations is tested in patients and controls. A regression analysis including the different EPICOG-SCH subtests at baseline as predictors of functionality at the different time points to explore the best predictive algorithms at 1 and 3 years of patient’s functionality in daily life following a first-episode.

**Conclusions:**

The EPICOG-SCH brief battery is modelled to be a useful first to screen for the cognitive impact of schizophrenia in daily functionality daily life. This research work will validate the composite scores for a context of use of first-episodes schizophrenia and follow up. To date it has not been described an efficient and straightforward way for clinicians working with schizophrenia to transfer quantitative information about patient cognitive profile into real life. The EPICOG-SCH global composite score provides valuable information to clinicians which can facilitate disease management, drawing a roadmap for cognitive rehabilitation, and planning of supportive resources from the community and health care system.

**Disclosure of Interest:**

None Declared

